# Production of High-Value Nanoparticles via Biogenic Processes Using Aquacultural and Horticultural Food Waste

**DOI:** 10.3390/ma10080852

**Published:** 2017-07-25

**Authors:** Purabi R. Ghosh, Derek Fawcett, Shashi B. Sharma, Gerrard E. J. Poinern

**Affiliations:** 1 Murdoch Applied Nanotechnology Research Group, Department of Physics, Energy Studies and Nanotechnology, School of Engineering and Energy, Murdoch University, Murdoch, Western Australia 6150, Australia; P.Ghosh@murdoch.edu.au (P.R.G.); Fawcett@southwest.com.au (D.F.); 2 Department of Primary Industries and Regional Development, 3 Baron Hay Court, South Perth, Western Australia 6151, Australia; Shashi.sharma@agric.wa.edu.au

**Keywords:** nanoparticles, biogenic synthesis, green chemistry, recycling, adding value

## Abstract

The quantities of organic waste produced globally by aquacultural and horticulture are extremely large and offer an attractive renewable source of biomolecules and bioactive compounds. The availability of such large and diverse sources of waste materials creates a unique opportunity to develop new recycling and food waste utilisation strategies. The aim of this review is to report the current status of research in the emerging field of producing high-value nanoparticles from food waste. Eco-friendly biogenic processes are quite rapid, and are usually carried out at normal room temperature and pressure. These alternative clean technologies do not rely on the use of the toxic chemicals and solvents commonly associated with traditional nanoparticle manufacturing processes. The relatively small number of research articles in the field have been surveyed and evaluated. Among the diversity of waste types, promising candidates and their ability to produce various high-value nanoparticles are discussed. Experimental parameters, nanoparticle characteristics and potential applications for nanoparticles in pharmaceuticals and biomedical applications are discussed. In spite of the advantages, there are a number of challenges, including nanoparticle reproducibility and understanding the formation mechanisms between different food waste products. Thus, there is considerable scope and opportunity for further research in this emerging field.

## 1. Introduction

**Figure 1:**
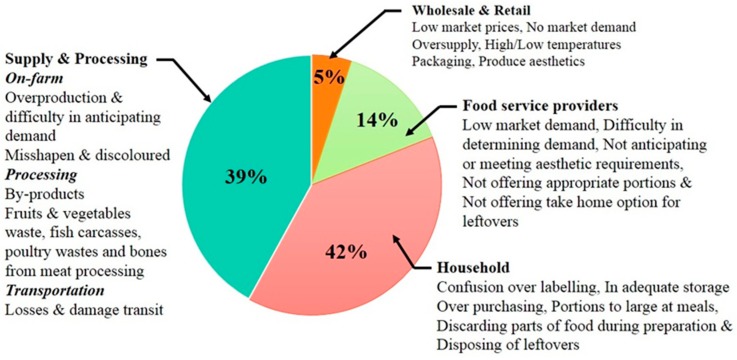
A summary of major sources of food waste from producer to household consumer [12,13,14].

Two decades ago aquacultural and horticultural organic waste was not considered a major economic cost or resource loss to food processing industries [1]. However, recent public concerns about hunger, conservation, environmental degradation and the socioeconomic impact of food waste have accelerated research into developing strategies that can reduce food waste and promote effective waste utilisation methodologies [2]. In addition, global concerns regarding the limited natural resources currently available and the ability to effectively use these resources to feed the predicted population of 12.3 billion in 2100 has accelerated research into finding more effective resource utilisation and management strategies [3,4,5]. Therefore, efficient and cost = effective strategies are needed to reduce organic waste and develop better food waste utilisation practices that can assist in the overall management of the food supply chain [6,7]. In the context of this review, aquaculture relates to the industrial sector involved in farming marine and marine capture, while the horticultural sector principally focuses on the production, processing and retail sales of fruits and vegetables to consumers. From a practical point of view, food processing will always produce a certain amount of waste. However, the waste currently being produced during food processing is disproportionate compared to other processing industries. For example, Buzby et al. estimated in 2014 the total value of food losses for three large and expensive agricultural waste streams at the retail and consumer levels in the United States. The streams and their respective costs included grains (US$36.1 billion), vegetables (US$108.7 billion) and fruits (US$62.2 billion) [8]. Similarly, many other developed countries have waste trends in their respective agricultural sectors. For instance, in 2014 Segre and Falasconi estimated the Italian agricultural sector left around 17.7 million tonnes or 3.25% of its total produce in the ground [9]. Factors contributing to waste generation include produce sizing, aesthetic standards, produce quality, production surpluses and marketing as seen in Figure 1. Annually, in the USA these factors have resulted in around 2.7 million tonnes of fruits and vegetables not being harvested or sold [10]. In developing countries like India, between 18% and 40% of all fresh fruits and vegetables grown end up as waste. This large amount of waste equates to an annual financial loss of around US$71,481 million to the Indian agro-food industry [11]. From a European perspective, studies carried out in the Netherlands indicate the annual cost of food waste to be around €4.4 billion (US$4.9 billion) [12]. Surprisingly, many studies have shown that the agro-food sector can produce waste levels that are typically around 39% of total production, as seen in Figure 1 [13,14,15].

From the marine perspective, macroalgae or seaweed are plant-like organisms that are routinely washed up on beaches and shorelines in large quantities around the world. Edible seaweeds are consumed in many parts of the world since they are highly nutritional foods that are rich in proteins and a source material for many medicinal remedies [16,17,18]. Seaweed is a staple food in daily use throughout South-East Asia, and the health benefits derived from its consumption have resulted in numerous studies investigating the medicinal and pharmaceutical uses of seaweed [1,19,20]. Several studies have revealed that seaweed is rich in antioxidants, carbohydrates, carotenoids, polysaccharides, polyunsaturated fatty acids, proteins, vitamins and also contains numerous secondary metabolites [21,22,23]. These naturally occurring biological compounds have been used in traditional Chinese medicine for centuries [19], and recently several seaweed based extracts have been used to complement conventional treatments and supplement alternative therapies [24,25,26]. Furthermore, studies have reported anti-inflammatory and inhibitory properties being exhibited by several seaweed extracts [27,28]. These medicinal properties have also been found to reduce blood pressure levels [29], reduce the incidence of cardiovascular diseases [30], and suppress some forms of cancer [31,32]. In 2013, Tacon and Metian estimated that around 95.5% (12 million tonnes) of the total global production of marine plants is supplied by aquaculture (farming of marine plants), while the remaining 4.5% (0.44 million tonnes) is made up of marine capture [33]. The majority of aquaculture production (around 9 million tonnes) is destined for human consumption. The remaining tonnage undergoes processing to extract phycocolloids, a highly nutritious ingredient that is added to farm animal and aquaculture feeds [33,34]. While aquatic plants are an important commodity in the global food supply chain, there is very little data in the literature reporting levels of waste generation, waste management strategies and disposal protocols [15].

A number of utilisation strategies for processing waste fruits, vegetables and grains have been investigated [35,36,37,38]. The aim of these strategies is to maximise the value and practical benefits from food waste, which will ultimately reduce the amount of waste going to landfill [39]. Unfortunately, reviewing the literature reveals few studies that assess the economic benefits of the various waste management strategies operating at a commercial scale. However, the literature does clearly identify agricultural waste as an important source of chemicals, bioactive compounds and pharmaceuticals [40]. Importantly, the current high demand for pharmaceutical ingredients, enzymes, solvents and surfactants has resulted in many countries developing strategies for converting agricultural waste into products for chemical feedstock. This alternative, biologically based approach provides a large variety of chemical compounds from a wide range of renewable agricultural waste for recycling and processing by both chemical and pharmaceutical industries. For example, succinic acid can be obtained from crop waste like sugarcane, maize, rice, barley and potatoes [41]. Similarly, starch-based plastic can be produced from cassava, maize and wheat [42], fatty acids can be generated from coconut and oil palm [43], and polymers, lubricants, adhesives, solvents, and surfactants can be derived from rapeseed and sunflower [44]. These studies clearly demonstrate that materials commonly thought of as waste are rich in biologically active compounds that can be used to produce high-value chemical and pharmaceutical products. Furthermore, recent studies have also shown that both aquacultural horticultural waste can be used as renewable feedstock for the manufacture of high-value nanoparticles [45,46,47]. Food waste and nanoparticle synthesis sounds like an unlikely combination, but recent investigations in the literature have shown that naturally occurring biomolecules present in waste have the potential to produce nanoparticles (particles less than 100 nm) with unique medicinal and pharmaceutical properties [48,49,50].

Both aquacultural and horticultural food waste contain beneficial biomolecules and compounds that can play an active role in reducing precursor metal ions in aqueous solutions to form nanoparticles. The biomolecules also act as modelling agents that direct particle growth in particular orientations, while other biomolecules act as capping agents to prevent nanoparticle agglomeration [45,51]. Another interesting feature of nanoparticles synthesised from food waste is the potential to deliver reliable, sustainable and green chemistry-based technologies that are eco-friendly, thus reducing the human health and environmental degradation risks normally associated with the use of toxic solvents and chemicals during conventional physical and chemical manufacturing protocols [52]. The considerable interest in nanoparticles shown by the scientific community is due to their unique physiochemical properties and their application in a number of fields such as pharmaceuticals and biomedicine. The extremely small size and large surface area to volume ratio are two material features that give nanoparticles their new or enhanced properties compared to conventional bulk forms of the same substance [53]. For example, gold (Au) nanoparticles are already widely used in medicine for diagnostics [54,55,56], targeted delivery of pharmaceuticals [57,58,59,60] and tumour destruction via hyperthermia [61]. While silver (Ag) nanoparticles display a broad spectrum antimicrobial activity against many human and animal pathogens [62,63,64,65] and as a result are used as antimicrobial agents in a wide range of commercially available medical and consumer products [66,67,68], both platinum (Pt) and palladium (Pd) nanoparticles have been used as catalysts [69,70,71]. Furthermore, metal oxide nanoparticles such as copper oxide (Cu_2_O, CuO) and zinc oxide (ZnO) have displayed antimicrobial activity [72,73] and because of this ZnO has been used in a variety of food packaging applications [74]. Moreover, super-paramagnetic ferric oxide (Fe_3_O_4_) nanoparticles have the potential to be used in a wide variety of biomedical applications such as magnetic resonance imaging, hyperthermia treatments and as a carrier for anti-cancer drugs [75,76,77]. The utilisation of aquacultural and horticultural wastes for the biogenic synthesis of high-value products such as metal and metal oxide nanoparticles is a fairly new field of research. Accordingly, only a relative few articles have appeared in the literature reporting the use of various aquacultural and horticultural waste to produce nanoparticles using eco-friendly green chemistry-based technologies [78,79,80,81]. The present work summarises current research in this relatively new research field and discusses the various experimental parameters that govern nanoparticle formation and growth during biogenic synthesis. The remainder of the review discusses the potential applications of aquacultural and horticultural food-waste produced nanoparticles in fields such as pharmaceuticals and biomedicine.

## 2. Biogenic Synthesis of Nanoparticles Using Aquacultural and Horticultural Food Waste

The biogenic synthesis of a variety of metal and metal oxide nanoparticles using aquacultural and horticultural food waste can be considered an alternative, eco-friendly and viable green chemistry-based route. In recent years there have been numerous studies reporting the synthesis of nanoparticles using a diverse array of plants sources. However, relatively few studies have reported using aquacultural and horticultural food waste to produce value-added products such as nanoparticles. Like the plant sources reported in the literature, aquacultural and horticultural waste also contains a vast array of biomolecules such as alkaloids, amino acids, enzymes, phenolics, proteins, polysaccharides, saponins, tannins, terpinoids and vitamins that can all be used to assist in the creation of nanoparticles [82]. The biogenic synthesis of nanoparticles is a bottom-up approach, during which atoms and molecules combine to form precursor building blocks that subsequently self-assemble [83]. Reviewing the various studies reported in the literature, it is evident that plant-based synthesis procedures have successfully produced a variety of noble metal nanoparticles such as gold, silver, platinum and palladium [45]. A few studies have also reported the formation of noble metal nanoparticles from extracts taken from aquacultural and horticultural food waste. For instance, Dubey et al. have reported the formation of 16-nm Ag spheres and 11-nm Au nano-triangles when *Tanacetum vulgare* (tansy fruit) extract was allowed to react with aqueous solutions of AuCl_4_^−^ ions and Ag^+^ ions respectively [84]. In addition, several other food waste extracts such as *Pyrus* sp. (pear fruit) and *Mangifera indica* (mango peel) have demonstrated their ability to reduce Au (III) ions to form Au nanoparticles [49,85]. *Citrus sinensis* (orange peel) and *Ananas comosus* (pineapple) extracts have shown the ability to reduce Ag^+^ ions in aqueous solutions to form Ag nanoparticles [78,86]. Likewise, Pt and Pd nanoparticles have been synthesised using *Musa paradisiac* (banana peel), tea and coffee extracts, and lignin [71,87,88]. 

**Figure 2:**
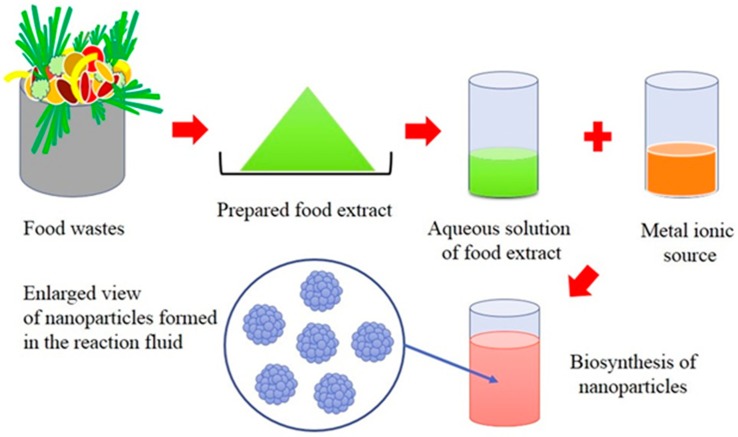
Schematic representation of the biogenic synthesis of nanoparticles using aquacultural and horticultural food waste extracts.

**Figure 3:**
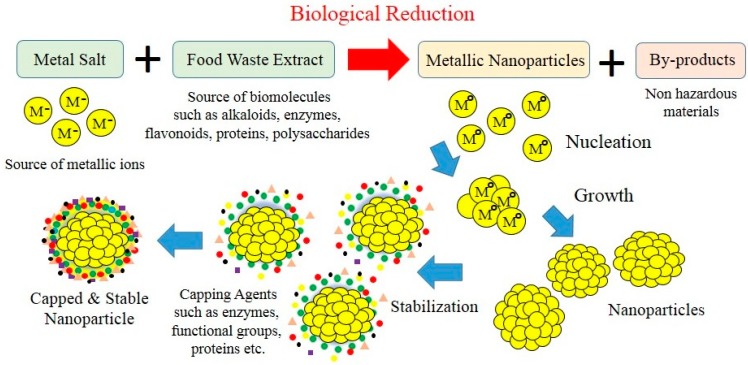
Schematic diagram of the mechanism behind the biogenic synthesis of metallic nanoparticles.

The advantage of using aquacultural and horticultural food waste is that it is readily available. This makes them a renewable feedstock that creates an alternative waste utilisation strategy for manufacturing high-value metal and metal oxide nanoparticles. Importantly, food waste offers a green, chemistry-based route that is rapid, cost-effective and eco-friendly. Metal nanoparticle production is a straightforward room temperature process that begins by mixing an aqueous metal salt solution with an aqueous solution containing a food waste extract as seen in Figure 2. Biogenic reduction starts immediately, and as the reduction process continues there is a distinctive colour change in the reaction mixture, indicating nanoparticle formation. For instance, a recent study by Kaviya et al. reported the formation of Ag nanoparticles when *Citrus sinensis* (orange) peel extract was used as the reducing agent. Reduction of Ag nanoparticles occurred within 20 min and their formation was clearly indicated by the reaction mixture changing colour from colourless to a yellowish-brown. Subsequent characterisation of the samples revealed nanoparticle morphology was spherical and their size was heavily dependent on reaction mixture temperature. At 25 °C the mean particle size was 35 ± 2 nm, while at 60 °C size was 10 ± 1 nm [89]. Studies indicate the fundamental nanoparticle formation mechanism created during biogenic synthesis begins with metal ions in solution transforming from their mono- or divalent oxidation states to from zero-valent states as seen in Figure 3. Biomolecules present in the food waste extract initiates metal ion reduction and then promotes nucleation [90,91,92]. Progressively, smaller neighbouring particles start assembling on their low energy faces that ultimately results in thermodynamically stable nanoparticle formation. During this stage food waste biomolecules also act as natural surfactants (capping agents), which influence the orientation and assembly of the smaller particles during subsequent growth as schematically presented in Figure 3. The modelling action produced by surfactants during biological synthesis explains why growth occurs along preferential planes [93]. These preferential growth planes result in morphologies such as spheres, cubes, triangles, hexagons, pentagons and wires being formed [82,94]. The number of experimental parameters known to govern the nanoparticle formation mechanism include: (1) the nature of the food waste extract; (2) concentration of food waste in the reaction mixture; (3) metal ion concentration in the source solution; (4) reaction mixture pH; (5) reaction mixture temperature; and (6) contact time. All these parameters are important and can directly influence nanoparticle formation and subsequently their physiochemical properties [95].

An important issue that has arisen in recent years is the possible adverse health effects produced by nanometre-scale materials. Studies have shown the physiochemical properties of nanometre scale materials of the same material can vary due to differences in parameters such as particle size, aggregation, chemical reactivity, concentration, dispersion and morphology [96,97]. Even small changes in these parameters can significantly influence nanoparticle behaviour and their subsequent interactions in particular environments. Generally, toxicity issues arise from the deposition of hazardous chemicals and solvents on the surface of the nanoparticles during many conventional physical and chemical manufacturing processes. The removal of these hazardous materials is extremely difficult and their presence can induce significant toxicological and inflammatory responses if used in biomedical applications [98]. Moreover, naked nanoparticles do not exist very long in the physiological environment of the human body and biomolecules such as proteins rapidly attach to their surface, forming a corona [99]. Therefore, synthesising biocompatible nanoparticles via food waste extracts offers a greener, less toxic and eco-friendly approach that avoids the use of toxic chemicals and solvents commonly used in conventional manufacturing. Furthermore, aquacultural and horticultural food waste is a renewable and relatively inexpensive feedstock. However, before aquacultural and horticultural food waste can be used commercially to manufacture high-value nanoparticles there needs to be more research into resolving a number of shortcomings. These shortcomings include: (1) developing an all-inclusive nanoparticle formation mechanism; (2) investigate the influence of experimental parameters on nanoparticle size, shape and dispersion, and (3) refine the biosynthesis process to improve reproducibility [100,101,102]. In terms of commercialisation: (1) develop technologies that overcome the limitations of scaling up the biosynthesis process; (2) developing a continuous supply route for suitable aquacultural and horticultural food waste; and (3) optimise the waste management chain to fully utilise the biomolecules and bioactive chemicals present in aquacultural and horticultural food waste [1,13,103].

## 3. Types of Nanoparticles Produced by Aquacultural and Horticultural Food Waste

The biogenic synthesis of metal and metal oxide nanoparticles via aquacultural and horticultural food waste is a new and emerging field of study. Food waste has the potential to produce a wide range of particle sizes and shapes using green, chemistry-based techniques [82,103,104]. At present, only a relatively small number of articles have appeared in the literature reporting the use of aquacultural and horticultural food waste being used to synthesise nanoparticles. The following sections summarise and discuss the current state of research as reported in the literature. A selection of recent studies reporting the biogenic synthesis of metal and metal oxide nanoparticles using various horticultural food waste products are summarised and presented in Table 1. Table 2 presents a selection of metal and metal oxide nanoparticles synthesised using a variety of marine alga (seaweeds) commonly produced by aquaculture.

### 3.1. Silver (Ag) Nanoparticles

The diversity of aquacultural and horticultural food waste has led a number of researchers to investigate their potential use in synthesising a variety of metal nanoparticles. In particular, Ag nanoparticles have paved a way into exploring this new field due to the exceptional antimicrobial properties displayed by Ag compounds. For centuries, Ag has been used as an antimicrobial agent in numerous medicinal preparations. In recent years Ag nanoparticles, with their unique physiochemical and enhanced antimicrobial properties, have been incorporated into a variety of biomedical protocols and pharmaceuticals [105,106]. The reason for these superior antimicrobial properties comes from the Ag nanoparticles ability to cause cell membrane damage and toxicological damage to cellular DNA [107,108]. Recently, several studies have reported the reduction of Ag^+^ ions in aqueous solutions containing aquacultural and horticultural food waste. For example, the formation of spherical Ag nanoparticles ranging in size from 5 to 35 nm was reported by Ahmad and Sharma using an extract taken from *Ananas comosus* (Pineapple) [79]. Similarly, spherical-shaped crystalline Ag nanoparticles ranging in size from 3 to 12 nm were synthesised by Konwarha et al. using an extract taken from *Citrus sinensis* (Orange) peel [86] and spherical Ag nanoparticles ranging in size from 5 to 20 nm have been produced using an extract taken from *Citrus unshiu* (Mandarin) peel by Basavegowda et al. [109]. In addition, Njagi et al. were able to use an aqueous solution containing *Sorghum* spp. (bran powder) extract to biologically synthesis spherical iron (Fe) and Ag nanoparticles that were typically around 10 nm in size [110]. Dubey et al. were able to synthesis both Ag and Au nanoparticles using *Tanacetum vulgare* (tansy fruit) [84]. Ag nanoparticles were spherical with a mean size of 16 nm and the Au nanoparticles were triangular plates that were typically around 11 nm in size. Likewise, Ankamwar et al. were able to use an *Emblica officinalis* (Indian Gooseberry) extract to synthesis both Ag and Au nanoparticles ranging in size from 10 nm to 25 nm [111]. Other researchers have investigated the use of marine algae, which are rich in polysaccharides, and other bioactive materials that can be used for synthesising nanoparticles. For example, Kannan et al. have reported the synthesis of Ag nanoparticles using *Codium capitatum* (seaweed). Their study revealed that two types of morphologies could be produced, namely spherical and cubic. Both morphologies ranged in size from 3 to 44 nm, with a mean particle size of 30 nm [112]. In a similar study by Castro et al., a green alga *Spyrogira insignis* was found to produce spherical Ag nanoparticles with a mean particle size of 30 nm [113]. Raeshkumar et al. have reported producing spherical Ag nanoparticles with a mean particle size of 14 nm using a brown seaweed, *Padina tetrastromatica* [114].

### 3.2. Gold (Au) Nanoparticles

Au nanoparticles are a very attractive material due to their wide application in fields such as catalytics, biomedicine, biosensors, pharmaceuticals, imaging and diagnostics [54,55,115,116,117]. Several recent studies have reported the reduction of aqueous chloroaurate solutions using a variety aquacultural and horticultural food waste products. For example, Krishnaswamy et al. have reported the formation of spherical Au nanoparticles ranging in size from 20 nm to 25 nm after using waste grape skins, stalks and seeds as the reducing agents [80]. Similarly, Ghodake et al. were able to synthesise triangular and hexagonal crystalline gold nanoparticles ranging in size from 200 nm to 500 nm using an extract taken from *Pyrus* sp. (pear) [85]. Likewise, Yang et al. have reported the formation of Au nanoparticles, ranging in size from 6.03 ± 2.77 nm to 18.01 ± 3.67 nm, using an extract taken from *Mangifera indica* (mango) peel [49]. Recently, Sharma et al. have produced Au nanoparticles using freshwater green alga (*Prasiola crispa*) and red alga (*Lemanea fluviatilis*) [118,119]. Studies have also shown that the type and concentration of biomolecules present in food waste can influence nanoparticle formation and their subsequent stability. A study by Huang et al. found that varying the concentration of sundried *Cinnamomum camphora* leaf extract or increasing the precursor chloroauric acid concentration in the reaction mixture resulted in nanoparticle shape changes (i.e., triangular to spherical) [120]. Similarly, Chandran et al. have reported varying the concentration of *Aloe vera* leaf extract in reaction mixtures containing chloroaurate ions to regulate nanoparticle size. The varying concentration not only regulated the size range between 50 and 350 nm, but also influenced the ratio of spherical to triangular nanoparticles produced [121]. Narayanan et al. were able to synthesis varying ratios of decahedral, hexagonal, triangular and spherical Au nanoparticles by changing the concentration of *Coleus amboinicu* leaf extract in the reaction mixture [122]. Furthermore, Ahmada et al. have reported that a low precursor Au concentration (1.53 mM) in a reaction mixture containing aqueous *Elaise guineensis* (oil palm) leaf extract produced spherical nanoparticles with a mean diameter of 27.89 ± 14.59 nm. However, a larger precursor Au concentration (4.055 mM) produced spherical, triangular, pentagonal and hexagonal nanoparticles with a mean particle diameter of 22.88 ± 8.21 nm [123]. The study also revealed a multilayer coating composed of carboxylic and phenolic compounds, which prevented the nanoparticles from agglomerating, thus indicating the importance of sufficient extract concentration to provide the necessary biomolecules needed for nanoparticle stabilization. 

### 3.3. Other Types of Nanoparticles

In recent years a few studies have reported the formation of several other types of nanoparticles produced from aquacultural and horticultural food waste. For example, Bankar et al. have produced palladium (Pd) nanoparticles using an aqueous solution containing an extract taken from *Musa paradisiac* (banana) peel. The resulting nanoparticles were crystalline and irregular in shape, and had a mean size of 50 nm [88]. Similarly, Lakshmipathy et al. used an extract taken from watermelon rind to form Pd nanoparticles with a mean particle size of 96 nm [124]. Coccia et al. were able to produce both Pd and platinum (Pt) nanoparticles using lignin [71]. In addition, Nadagouda and Varma were able to use commercially available tea and coffee waste extracts to produce both Ag and Pd nanoparticles. Their study found that both types of nanoparticles were spherical and ranged in size from 5 nm to 100 nm, with the majority of the particles falling within the 20 to 60 nm range [87]. Furthermore, a recent study by Lunge et al. found that waste tea extracts could produce magnetic ferric oxide (Fe_3_O_4_) nanoparticles. The nanoparticles formed ranged in size from 5 to 25 nm and consisted of both cubes and pyramids [125]. Moreover, a study by Mahdavi et al. that used extracts from brown seaweed (*Sargassum muticum*) could be used to form ferric oxide (Fe_3_O_4_) nanoparticles. Their study also identified the water-soluble polysaccharide cell walls that contained amino, carboxyl and hydroxyl functional groups as the biomolecules that acted as both reducing and capping agents. The resulting crystalline nanoparticles were cubic in nature and had a mean particle size of 18 ± 4 nm [126]. Also using a brown seaweed extract (*Bifurcaria bifurcata*), Abboud et al. were able to produce both cuprous oxide (Cu_2_O) and cupric oxide nanoparticles (CuO). Nanoparticle morphology was predominantly spherical, with particles ranging in size from 5 to 45 nm [72]. Similarly, Khanehzaei et al. were able to produce spherical Cu-cored Cu_2_O nanoparticles with a mean particle size of 53 nm using extracts taken from red seaweed (*Kappaphycus alvarezii*) [127]. Other types of metal oxide nanoparticles produced from extracts taken from *Citrus sinensis* (orange) peels and *Musa paradisiac* (banana) peels include magnesium oxide (MgO) and manganese (II, III) oxide (Mn_3_O_4_) respectively. For example, Rao et al. were able to produce spherical MgO nanoparticles with a mean particle size of 29 nm using extracts taken from *Citrus sinensis* (orange) peel [128]. In a similar study, Yan et al. were able to use *Musa paradisiac* (banana) peel extract to form spherical Mn_3_O_4_ nanoparticles ranging in size from 20 nm to 50 nm. Their study also identified the super-capacitive properties of the nanoparticles and their potential use in high-stability Mn_3_O_4_-based electrodes [129].

## 4. Applications and Future Perspectives

Metal and metal oxide nanoparticles produced using conventional physical and chemical manufacturing processes, as mentioned earlier, have been used in variety of antimicrobials products, biosensors, photo-catalysts, pharmaceuticals, cancer therapy and food packaging applications. Aquacultural and horticultural food waste extracts have the potential to become another avenue for the green synthesis of various types of metal and metal oxide nanoparticles as seen in Table 1 and Table 2. With the diversity of food waste available and the ability to influence the reaction parameters during synthesis, there are many opportunities to produce novel metal-based nanoparticles with unique properties. However, despite the great interest shown in the biogenic synthesis of nanoparticles using aquacultural and horticultural food waste, there have only been a few practical applications reported. In spite of this, studying the properties of conventionally manufactured nanoparticles and comparing their properties with those produced using aquacultural and horticultural food waste should provide an indication of property differences and potential applications. The property differences arise from the use of hazardous chemicals and solvents (capping agents and surfactants) commonly used in many conventional manufacturing processes and their non-use in green biogenic synthesis procedures. Thus, biosynthesised nanoparticles should have a wider range of biomedical applications since they are free from relatively hazardous chemicals and solvents [97,98].

Noble metal nanoparticles, in particular Ag and Au, produced using a variety of techniques, are currently used in a wide range of detection, imaging, diagnostics and therapeutic applications [146,147,148]. In particular, using Au nanoparticles to promote DNA damage in cancerous cells through a variety of targeted therapeutic treatments is currently being applied in several therapeutic procedures [115,149,150]. Furthermore, both Ag and Au nanoparticles have a broad spectrum of antimicrobial properties that have been tested against a several human and animal pathogens [151,152,153]. With regards to horticultural food waste extracts, Jain et al. have used *Carica papaya* (pawpaw) to biosynthesise Ag nanoparticles and then evaluate their antimicrobial properties against a number of pathogens [131]. Similarly, Shanmugam et al. have biosynthesised Ag nanoparticles using the marine seaweed *Sargassum wightii* and then verified the nanoparticles’ antimicrobial activity against a number of human pathogens [154]. Ag nanoparticles produced in both food waste studies were found to have similar antimicrobial properties to those synthesised by conventional manufacturing processes. This is of particular importance since Ag nanoparticles produced by traditional methods are currently being used as antimicrobial agents in a wide range of medical and consumer products [66,67,68,155]. Thus, biosynthesising Ag nanoparticles from food waste is an alternative technique that can produce nanoparticles with properties similar to those produced by more conventional methods. Similarly, Ag and Pd nanoparticles were produced using waste tea and coffee extracts by Nadagouda and Varma [87]. During synthesis the individual metal salts were first reduced and then capped by polyphenols present in the respective extracts. Interestingly, recent studies have also revealed that Pd nanoparticles produced using food waste can be used as catalysts. The catalytic behaviour of produced Pd nanoparticles has been studied using oxidation and reduction reactions [71,124]. Furthermore, a recent study by Petla et al. identified and quantified the catalytic properties of Pd nanoparticles produced using soya leaf extract by successfully degrading several azo dyes [156].

The biogenic synthesis of less noble metals and metal oxide nanoparticles have also attracted the interest of several researchers. At present there are serious concerns about antibiotic resistance, complex manufacturing protocols, environmental pollution, and the high manufacturing costs associated with conventional pharmaceutical preparations [157]. Therefore, in recent years there has been a search for natural antimicrobial agents that can overcome the shortcomings of currently available antimicrobial pharmaceuticals. For example, copper (Cu) and Cu oxide nanoparticles are natural antimicrobial agents that have been biosynthesised using a variety of plant-derived extracts. A study by Lee et al. found Cu nanoparticles produced by a leaf extract from *Magnolia Kobus* formed spherical particles ranging in size from 40 to 100 nm and displayed antimicrobial properties towards *Escherichia coli* [158]. From a marine perspective, Abboud et al. have biosynthesised copper oxide nanoparticles using a brown algal extract (*Bifurcaria bifurcata*). The resulting nanoparticles were spherical, ranged in size from 5 to 45 nm and displayed good antibacterial properties towards both *Enterobacter aerogenes* and *Staphylococcus aureus* [72]. Furthermore, Nagarajan and Kuppusamy have biosynthesised zinc oxide (ZnO) nanoparticles from a brown seaweed extract (*Sargassum myriocystum*) that displayed antimicrobial properties against a number of bacteria and fungi [159]. However, at present no studies have appeared in the literature reporting the used of food waste to biosynthesise ZnO nanoparticles. The abovementioned antimicrobial studies demonstrate that biogenic synthesis can be used as an alternative method for producing nanoparticles with the potential to assist in the management of infectious diseases caused by bacteria.

However, the biosynthesis of metal and metal oxide nanoparticles using aquacultural and horticultural food waste extracts is still in its infancy. Consequently, only a few oxides have been studied and reported. Those recently reported include ferric oxide (Fe_3_O_4_), magnesium oxide (MgO) and manganese (II, III) oxide (Mn_3_O_4_). As mentioned earlier, Lunge et al. have successfully used waste tea extracts to produce (Fe_3_O_4_) nanoparticles. Their studies found that these cubic and pyramidal nanoparticles could be effectively used to remove arsenic metal ions from aqueous solutions [125]. Other studies reporting the biosynthesis of (Fe_3_O_4_) nanoparticles have involved using brown seaweed (*Sargassum muticum*) extracts [126,160]. Namvar et al. have used brown seaweed to produce (Fe_3_O_4_) nanoparticles that were subsequently assessed via in vitro cytotoxicity and anticancer testing for their activity against human cell lines for leukaemia, breast cancer, cervical cancer and liver cancer [160]. The results of their studies revealed that the accumulation of (Fe_3_O_4_) nanoparticles in treated cells tended to promote cell apoptosis, which suggested that an alternative cancer treatment protocol is possible using these nanoparticles. In terms of other oxides, Ganapathi Rao et al. have reported the biogenic synthesis of MgO nanoparticles from orange fruit waste [128] and Yan et al. have reported producing Mn_3_O_4_ nanoparticles with super-capacitive properties using *Musa paradisiac* (banana) peel extract [129].

The present review has demonstrated that the use of aquacultural and horticultural food waste extracts can be used to manufacture metal and metal oxide nanoparticles. Currently, this new and emerging field has produced relatively few study articles. The review has also shown that this new field needs to be fully explored and the development of eco-friendly and efficient green chemistry-based methods for recycling and utilising food waste needs to take place. Both aquacultural and horticultural food waste are produced in extremely large quantities around the world and offer an attractive and renewable source of biomolecules and bioactive compounds. The availability of such a large and diverse source of food waste creates a unique opportunity to develop new recycling and food waste utilisation strategies. One such value-adding strategy reported in the present work is the use of food waste to produce high-value metal and metal oxide nanoparticles. These high-value nanoparticles have the potential to be used in a wide range of current and future medical and pharmaceutical products.

## 5. Conclusions

The recycling and utilisation of food waste produced from aquacultural and horticultural industries has several advantages. The first is that the extremely large quantities of waste generated globally offer an attractive and renewable source of biomolecules and bioactive compounds. Processing food waste using chemistry-based strategies is eco-friendly, can significantly reduce the amount going to landfill, and creates a wide range of value-added products. One such strategy, presented in this review, is the biogenic synthesis of metal and metal oxide nanoparticles using aquacultural and horticultural food waste. The green biogenic synthesis route has several advantages over more traditional nanoparticle manufacturing processes. The procedure is straightforward, can be scaled up, and is eco-friendly. The most attractive feature of the biogenic synthesis route is that it can produce nanoparticles free from the toxic non-biodegradable commercial chemicals and surfactants that are commonly used in many conventional physical and chemical manufacturing processes. Studies reported and discussed in the present work have demonstrated that aquacultural and horticultural food waste extracts can be used to manufacture a wide variety of nanoparticles free from toxic chemicals and surfactants, thus, making these nanoparticles ideal candidates for pharmaceutical products and biomedical applications. This is unlike traditional nanoparticle manufacturing processes, which often leave detrimental surface coatings (solvents and surfactants) that severely limit their use in many therapeutic applications. For example, Ag and Au nanoparticles produced by food waste were reported to have a broad spectrum of antimicrobial properties against several human and animal pathogens. Furthermore, food waste has been reported by several researchers to produce Cu, Cu oxides, and ZnO nanoparticles. These nanoparticles were all found to display antimicrobial properties against several bacteria and fungi, while Fe_3_O_4_ nanoparticles synthesised using seaweed have displayed anticancer activity against a number of human cell lines. However, despite the many advantages of using food waste extracts to generate nanoparticles, there are a number of unresolved issues that need to be elucidated—for example, the variation of nanoparticle size and shape reproducibility when using different food waste extracts. Furthermore, there are also issues involving variations in formation mechanisms between different food waste products that need to be resolved. At present, only a few studies have reported using food waste to generate nanoparticles. The limited number of studies creates considerable scope and opportunity for further research in this emerging field.

## Figures and Tables

**Table 1:** A selection of nanoparticles biosynthesised by horticultural waste sources.

Nanoparticle	Size & Morphology	Food Source	Year	Reference
Ag	5 to 35 nm, Spherical	*Ananas comosus* (Pineapple)	2012	[79]
Ag & Au	Ag: 16 nm, Spherical, Au: 11 nm, triangular	*Tanacetum vulgare* (tansy fruit)	2010	[84]
Ag	3 to 12 nm, Spherical	*Citrus sinensis* (orange) peel	2011	[86]
Ag	35 ± 2 nm @ 25 °C, Spherical 10 ± 1 nm @ 60 °C, Spherical	*Citrus sinensis* (orange) peel	2011	[89]
Ag	5 to 20 nm, Spherical	*Citrus unshiu* (mandarin) peel	2013	[109]
Ag	10 nm, Quasi-spherical	*Sorghum* spp. (bran) (aw)	2010	[110]
Ag & Au	Ag: 10 nm to 20 nm, Spherical Au: 15 nm to 25 nm, Spherical	*Emblica officinalis* (Indian Gooseberry)	2005	[111]
Ag	Large nanoclusters	*Musa paradisiac* (banana) peel	2010	[88]
Ag	60 to 80 nm, Spherical	*Carica papaya* (pawpaw)	2008	[130]
Ag	15 nm, Cubic	*Carica papaya* (pawpaw)	2009	[131]
Ag	0.1 µm to 0.5 µm, Granular	*Psidium guajava* (guava)	2014	[132]
Ag	4.32 nm to 17.65 nm, Spherical	*Daucus carrota* L. (Black Carrot)	2014	[133]
Ag	4 nm to 22 nm, Spherical	*Allium sativum* (garlic clove)	2011	[134]
Ag	17.96 ± 0.16 nm, Spherical	*Citrullus lanatus* rind	2017	[135]
Au	20 to 140 nm, Spherical	*Citrullus lanatus* rind	2015	[136]
Au	20 to 25 nm, Quasi-spherical	Grape skin, stalk and seed waste	2014	[80]
Au	50 to 100 nm, Spherical	Rice bran (aw)	2014	[81]
Au	200 to 500 nm, Triangular, hexagonal	*Pyrus* sp. (pear)	2010	[85]
Au	6.03 ± 2.77 to 18.01 ± 3.67 nm, Spherical	*Mangifera indica* (mango) peel	2014	[49]
Au	432.3 nm, Shape not specified	*Daucus carota*, subsp. *Sativus* (Carrot)	2014	[137]
Au	Micro-scale, Triangular	*Cicer arietinum* L. (Bean extract)	2006	[138]
Au	pH 9: 10 nm, Spherical, pH 10: 25 nm, Spherical, rods, pH 11: 15 nm diameter nanowires of varying length	*Beta vulgaris* (sugar beet pulp)	2011	[139]
Pd	50 nm, Crystalline, irregular shape	*Musa paradisiac* (banana) peel	2010	[88]
Pd & Ag	20 nm to 60 nm, Spherical	Various commercially available tea/coffee extracts	2008	[87]
Pd & Pt	16 to 20 nm, Spherical	Lignin (aw)	2012	[71]
Pd	96 nm, Spherical	*Citrullus lanatus* (watermelon) rind	2015	[124]
Fe_3_O_4_	5 to 25 nm, Cubes & Pyramids	Tea Waste	2014	[125]
MgO	29 nm, Spherical	*Citrus sinensis* (orange) peel	2015	[128]
Mn_3_O_4_	20 nm to 50 nm, Spherical	*Musa paradisiac* (banana) peel	2014	[49]

Note: (aw) indicates agricultural waste.

**Table 2:** A selection of nanoparticles biosynthesised using marine plant sources.

Nanoparticle	Size & Shape	Marine Alga	Year	Reference
Ag	3 to 44 nm, Spherical and Cubic	*Codium capitatum*	2013	[112]
Ag	30 nm, Spherical	*Spyrogira insignis*	2013	[113]
Ag	4 to 24 nm, Spherical	*Enteromorpha compressa*	2017	[140]
Ag Au	20 nm, Spherical 5 to 260 nm, Triangles, Spheres and Hexagons	*Sargassum incisifolium*	2016	[141]
Au	6 to 10 nm, Spherical & Triangular	*Turbinaria conoides*	2013	[142]
Au	18.7 to 93.7 nm, Spherical	*Stoechospermum marginatum*	2012	[143]
Pd	4 to 6 nm, Spherical	*Laminaria digitata*	2015	[144]
Cu_2_O, CuO	5 to 45 nm, Spherical	*Bifurcaria bifurcata*	2014	[72]
Cu/Cu_2_O	53 nm, Spherical	*Kappaphycus alvarezii*	2014	[127]
Fe_3_O_4_	18 ± 4 nm, Cubic	*Sargassum muticum*	2013	[126]
ZnO	18 to 50 nm, Hexagonal	*Gracilaria gracilis*	2014	[145]
